# RIP3 dependent NLRP3 inflammasome activation is implicated in acute lung injury in mice

**DOI:** 10.1186/s12967-018-1606-4

**Published:** 2018-08-20

**Authors:** Jingxian Chen, Shuang Wang, Rong Fu, Mianjing Zhou, Tengyue Zhang, Wenxu Pan, Niansheng Yang, Yuefang Huang

**Affiliations:** 1 Department of Pediatrics, First Affiliated Hospital, Sun Yat-sen University, 58 Zhongshan Road II, Guangzhou, 510080 People’s Republic of China; 2 Department of Rheumatology, First Affiliated Hospital, Sun Yat-sen University, Guangzhou, 510080 People’s Republic of China; 3 Department of Rheumatology, Second Affiliated Hospital, Guangzhou Medical University, Guangzhou, 510260 People’s Republic of China

**Keywords:** Acute lung injury, RIP3, Necroptosis, Inflammasome, IL-1β

## Abstract

**Background:**

NLRP3 inflammasome is involved in the inflammatory responses during acute lung injury (ALI). RIP3 triggered NLRP3 inflammasome activation independent of necroptosis induction has recently been documented. In this study, the role of RIP3 in the activation of NLRP3 inflammasome in the development of ALI was investigated.

**Methods:**

A selective RIP3 inhibitor GSK872 was used to investigate the roles of RIP3 in NLRP3 inflammasome activation in the lipopolysaccharide (LPS) induced ALI mouse model. The mechanism of NLRP3 inflammasome activation was investigated in the human monocytic cell line THP-1. NLRP3 inflammasome and necroptosis were measured by flow cytometry or western blot. RIP3–NLRP3 interaction was interrogated using immunoprecipitation and the Duolink^®^ In situ detection.

**Results:**

Significant upregulation of both necroptosis and NLRP3 inflammasome pathways were observed in the lungs of mice with LPS induced ALI. GSK872 significantly suppressed the activation of necroptosis and NLRP3 activation with reduction of IL-1β and IL-18 production and inflammatory cells infiltration, resulting in a significant amelioration of lung injury. These two processes were shown to be active in interstitial macrophages and CD11b^+^ monocyte–macrophages/dendritic cells. In THP-1 cells, RIP3 and NLRP3 interaction was enhanced by LPS/ATP stimulation resulting in IL-1β and IL-18 production. This RIP3–NLRP3 interaction was significantly inhibited by GSK872.

**Conclusion:**

Taking together, these results show that RIP3 participates in the NLRP3 inflammasome activation in infiltrating macrophages in ALI induced by LPS. This process plays a significant pathogenic role in LPS-induced lung injury.

**Electronic supplementary material:**

The online version of this article (10.1186/s12967-018-1606-4) contains supplementary material, which is available to authorized users.

## Background

Acute lung injury (ALI) is a critical syndrome predisposed to acute respiratory distress syndrome (ARDS) and results in high morbidity and mortality [1]. Irrespective of its causes, inflammation mediated by innate immunity plays a pivotal role in the pathophysiology of ALI. We have shown that NLRP3 activation is implicated in the pathogenesis of ALI [2]. The activation of NLRP3 inflammasome results in the activation of caspase-1. Active caspase-1 cleaves the preformed IL-1β and IL-18 into their mature and active forms which participate in the inflammatory process in ALI [3].

Recently, considerable interest has been focused on necroptosis, a cellular processes characterized by RIP1/RIP3 phosphorylation [4]. The phosphorylated RIP1/RIP3 complex then interacts with a downstream molecule mixed lineage kinase domain-like protein (MLKL), which often results in cell death [5]. Necroptosis is accompanied by intensification of inflammation with the activation of NLRP3 inflammasome [6]. It was assumed that the RIP1–RIP3–MLKL necroptosis pathway was responsible for the inflammasome activation [6]. However, recent evidence shows that RIP3 activates NLRP3 inflammasome independent of the necroptosis pathway [7]. Thus RIP3 participates in two independent cellular processes.

RIP3-deficient mice had reduced inflammation in LPS-induced ARD [8]. This observation led to the conclusion that RIP3 mediated necroptosis contributed to the pathogenesis of LPS-induced ARDS. In view of dual function of RIP3 as discussed, we decided to interrogate these two processes in the LPS-induced ALI model to clarify the role of RIP3 dependent NLRP3 inflammasome activation during the development of ALI.

## Methods

### Mice

Male C57BL/6 mice were purchased from the Experimental Animal Center at Guangzhou University of Chinese Medicine (Guangzhou, China) and were housed under a pathogen-free condition in the Experimental Animal Center at Sun Yat-sen University, Guangzhou, China. The mice were cared for and the experiments were performed in accordance with the National Institutes of Health Guide for Care and Use of Animals. The experiments were approved by the Ethics Committee of Sun Yat-sen University.

### LPS-induced acute lung injury (ALI) model

LPS-induced ALI model was created as described previously [2]. Briefly, 8-week-old C57BL/6 mice were anesthetized with intraperitoneal ketamine (80 mg/kg) and xylazine (15 mg/kg). Six milligram per kilogram of LPS (Sigma-Aldrich St. Louis, MO, USA) was delivered to the lungs via a 20-gauge angiocath catheter. The control (sham operated) mice were given intratracheal PBS.

### Pharmacological blockage of RIP3

GSK872 (Merck Millipore, Damstadt, Germany), a selective RIP3 inhibitor was used. Mice were treated intraperitoneally with or without GSK872 (5 mg/kg) every 24 h while the first injection began at 2 h before LPS administration. Groups of 10 mice were sacrificed 48 h after LPS administration. The bronchoalveolar lavage (BAL) fluid was collected for cell count, protein quantification and enzyme-linked immunosorbent assay (ELISA). Lung tissues were collected for isolation of single cells, histology, ELISA and western blot analysis.

### Cell culture and activation of NLRP3 pathway

A human monocyte cell line THP-1 was purchased from the American Type Culture Collection (ATCC, Rockville, MD, USA) and cells were cultured at 37 °C, 5% CO_2_, RPMI 1640 (Life Technologies, Grand Island, NY, USA) containing 10% fetal calf serum (FCS) (Gibco, McHenry, MD, USA), 100 U/ml penicillin and 100 mg/ml streptomycin. THP-1 cells were primed with 1 μg/ml LPS in the presence or absence of GSK872 (5 mM) for 4 h followed by stimulation with ATP (5 mM) for 1 h. Cell supernatants were collected for detection of IL-1β (R&D Systems, Minneapolis, MN, USA) and IL-18 (Cusabio, Wuhan, China) by ELISA. Cells were subjected to flow cytometry (FACS), western blot and protein interaction analysis.

### Bronchoalveolar lavage (BAL)

BAL was performed as previously described [2]. Mice were anesthetized and sacrificed by heart puncture after opening the thoracic cavity. The trachea was exposed and an 18G sterile needle with blunt end was inserted into the trachea through a small semi-excision. PBS was injected and withdrawn for lavage. A total volume of 2.4 ml BAL fluid per mouse was collected. Supernatants were collected for ELISA and total protein analysis. Total cell counts of pelleted cells were determined on a grid hemocytometer. Total protein level was determined by using BCA Protein Assay Kit (Thermo Fisher Scientific, Grand Island, NY, USA) according to the manufacturers’ instructions.

### Preparation of single cell suspensions and flow cytometry (FACS)

Lung cell isolation was performed as previously described [9]. Lung single cell suspensions were stained with APC-conjugated anti-mouse CD45, PE-conjugated anti-mouse CD103, PerCP-Cy5.5-conjugated anti-mouse CD24 (all from eBioscience, San Diego, CA, USA), Alexa Flour 700-conjugated anti mouse I-A/I-E, BV421-conjugated anti-mouse CD11b (both from Biolegend, San Diego, CA, USA), PE-Cy7-conjugated anti-mouse CD11c, Alexa Flour 647-conjugated anti-mouse siglecF (both from BD Pharmingen, San Diego, CA, USA). Activation of NLRP3 inflammasome was detected as active caspase-1 level using FAM-FLICA Caspase-1 Assay Kit (Immunochemistry Technology, Bloomington, MN, USA). All FACS analyses were performed on a Gallios Flow Cytometer (Gallios, Beckman Coulter, Brea, CA, USA). Alveolar macrophages, interstitial macrophages (IMs), CD11b^+^ monocyte–macrophages/dendritic cells (M–M/DCs) cells and CD103^+^ DCs in lung tissues were isolated using the gating strategy reported by Misharin et al. and Kopf et al. [10, 11].

### Histology

After sacrifice, lungs were collected and fixed in 10% neutral formalin for 24 h. Lung tissues were embedded in paraffin and sectioned (2 μm), followed by hematoxylin and eosin (HE) staining.

### Western blot analysis

Proteins from lung tissues and THP-1 cells were extracted and analyzed by western blotting as described previously [12]. Total proteins were extracted with cell lysis buffer (Cell Signaling Technology, USA) according to the manufacturer’s instructions. Nuclear and cytosolic proteins were obtained with a commercial nuclear extraction kit (Thermo, USA) according to the manufacturer’s instructions. The primary antibodies used in this study included: mouse anti-NLRP3, mouse anti-caspase-1 (p20) (both from AdipoGen, San Diego, CA, USA), rabbit anti-RIP3, mouse anti-p-RIP3, rabbit anti-MLKL, rabbit anti-p-MLKL (all from Abcam, Cambridge, UK), mouse anti-RIP1 (R&D Systems, Minneapolis, MN, USA), rabbit anti-phospho-p44/42 MAPK kinase (p-ERK), total p44/42 MAPK kinase (t-ERK) and fibrillarin (all from Cell Signaling Technology, Beverly, MA, USA), and rabbit anti-GAPDH and anti-NF-κB p65 antibodies (both from Santa Cruz Biotechnology, Dallas, Texas, USA). HRP conjugated anti-mouse and anti-Rabbit IgG (both from Cell Signaling Technology, Beverly, MA, USA) were used as secondary antibodies. Signals were detected with enhanced chemiluminescence analysis kit (Cell Signaling Technology, Beverly, MA, USA).

### Immunofluorescence

THP-1 cells were fixed in 4% paraformaldehyde for 10 min, permeabilized in 0.01% Triton X-100 for 10 min and blocked in 5% BSA for 1 h. For p-RIP3 and p-MLKL staining, cells were incubated with mouse anti-p-RIP3 and rabbit anti-p-MLKL (both from Abcam, Cambridge, UK), overnight at 4 °C and then labeled with secondary anti-mouse antibody conjugated with Alexa Fluor 488 and anti-rabbit antibody conjugated with Alexa Fluor A555 (both from Thermo Fisher Scientific, Grand Island, NY, USA) respectively for 60 min, then mounted in Vectorlabs mounting media with 4′,6-diamidino-2-phenylindole (DAPI). Slides were visualized using a fluorescence microscope LSM 800 (Zeiss, Jena, Germany).

### Protein interaction studies

Detection of RIP1–RIP3 and RIP3–NLRP interaction in THP-1 cells were performed using the Duolink^®^ In situ Detection Reagents (Sigma-Aldrich, St. Louis, MO, USA) according to the manufacturers’ instructions. Briefly, THP-1 cells were first incubated with two primary antibodies that recognize target proteins (anti-RIP3 and anti-NLRP3 antibodies or anti-RIP1 and anti-RIP3 antibodies, all from Abcam, Cambridge, UK), and then incubated with a pair of proximity ligation assay (PLA) probes which is composed of species-specific secondary antibodies conjugated to complementary oligonucleotides. In the presence of hybridization solution and ligase, the oligonucleotides form a circle in case of close proximity of proteins. Finally, the polymerase and nucleotides participate to the formation of the rolling circle amplification, which were visualized in green fluorescence.

Immunoprecipitation (IP) was performed using the Thermo Scientific Pierce co-IP kit (Thermo Fisher Scientific, Grand Island, NY, USA) following the manufacturer’s protocol and conducted as previously described [13]. Briefly, THP-1 cells were washed with ice-cold PBS and lysed. The cell lysates were centrifuged, and the supernatant was subjected to IP with rabbit anti-RIP3 coated resin at 4 °C overnight. After the IP, the resin was washed three times and the IP proteins were subsequently analyzed for RIP1, RIP3, and NLRP3 by western blot.

### RNA interference

Small interfering RNA duplexes (si-RIP3) targeting the RIP3 (ID11035) (si-RIP3, Ribobio, Guangzhou, China) were synthesized for cell treatment. The THP-1 cells were transfected with 100 nmol siRNA or scramble siRNA and cultured for 48–72 h before stimulation.

### Statistical analysis

Data were presented as mean ± SEM. Statistical analyses were performed using one-way ANOVA. All data were analyzed using SPSS software (version 17.0). p values < 0.05 were considered statistically significant.

## Results

### Necroptosis and NLRP3 inflammasome pathways were activated in ALI

**Fig. 1:**
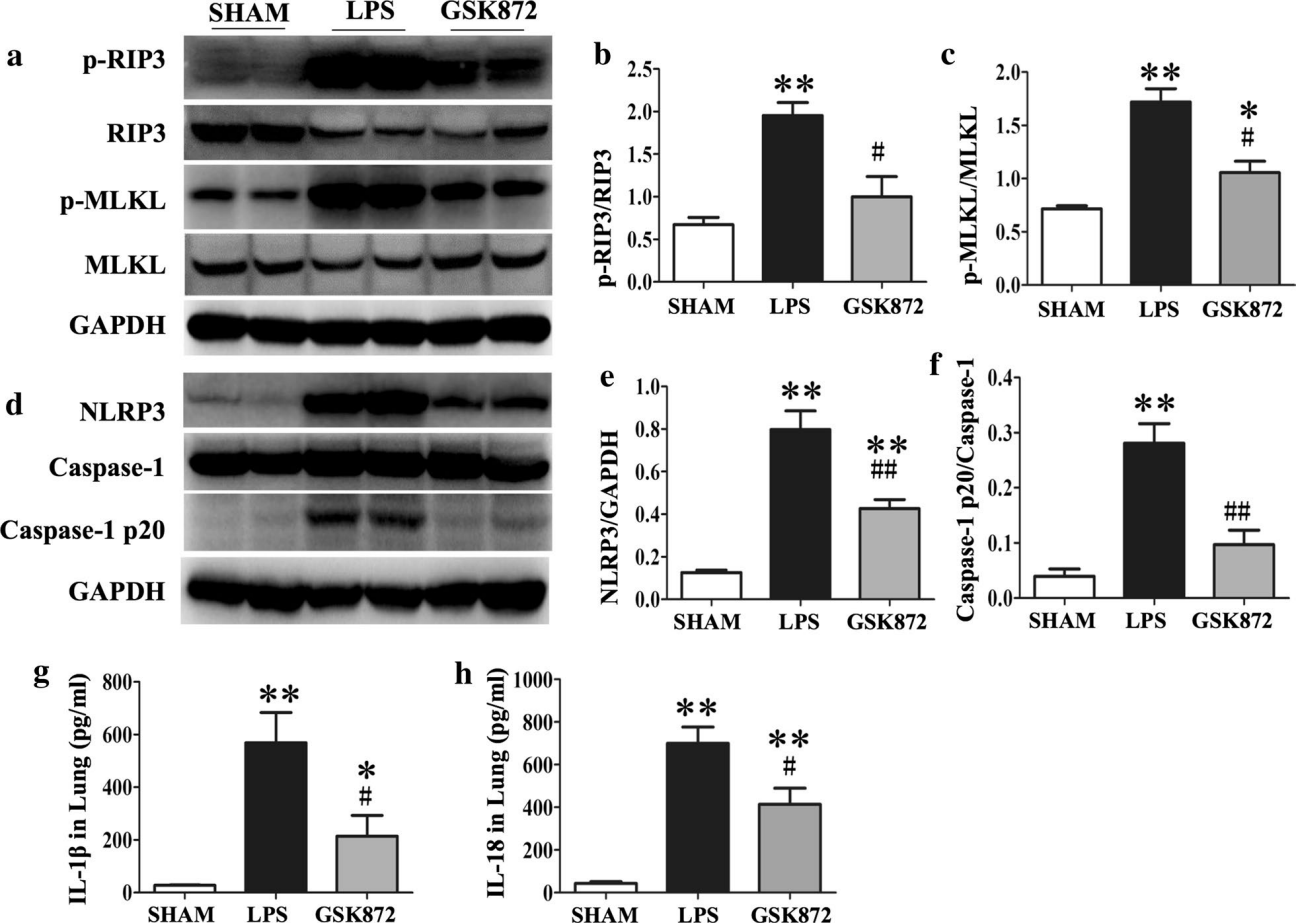
GSK872 suppressed RIP3 mediated necroptosis and activation of NLRP3 pathway in LPS-induced ALI mice. The RIP3 mediated necroptosis was detected by expression of p-RIP3, RIP3, p-MLKL and MLKL. The NLRP3 inflammasome activation was evaluated by expression of NLRP3, caspase-1 and caspase-1p20. **a** Representative western blot bands showed the protein expression of p-RIP3, RIP3, p-MLKL, MLKL and GAPDH in the lung tissues at day 2 after LPS induction. **b**, **c** Quantitative data showed protein expressions of p-RIP3 and p-MLKL normalized to the values of RIP3 and MLKL respectively. **d** Representative western blot bands showed the protein expression of NLRP3, Caspase-1, Caspase-1p20 and GAPDH in the lung tissues at day 2 after LPS induction. **e**, **f** Quantitative data showed protein expressions of NLRP3 and caspase-1p20 normalized to the values of GAPDH and caspase-1 respectively. **g**, **h** IL-1β and IL-18 level in lung tissues measured by ELISA. Each bar represents mean ± SEM (n = 6). *p < 0.05, **p < 0.01 versus sham mice; ^#^p < 0.05, ^##^p < 0.01 versus LPS-induced lung injury mice

Both RIP3 necroptosis pathway and NLRP3 inflammasome pathways were examined in the mouse model of LPS-induced acute lung injury (ALI). RIP3 mediated necroptosis pathway was significantly activated as demonstrated by enhanced expression of p-RIP3, p-MLKL in the lungs of mice with LPS-induced ALI compared with those of mice treated with PBS (Fig. 1a–c). Enhanced protein expression of NLRP3 and caspase-1p20 subunit, which represents the highly active p20/p10 tetrameric forms of processed caspase-1, were also observed in ALI mice. However, there were no significant changes in the expression of caspase-1 (Fig. 1d–f).

### Inhibition of RIP3 suppressed activation of necroptosis and NLRP3 pathway in ALI

Treatment of mice with LPS-induced ALI by a selective RIP3 inhibitor GSK872 resulted in the significant suppression of the activation of necroptosis pathway. Expression of p-RIP3/RIP3 and p-MLKL/MLKL in the lung tissues was significantly downregulated by GSK872 treatment as demonstrated by western blot analysis (Fig. 1a–c). Interestingly, the expression of NLRP3 and caspase-1p20/pro-caspase1 were also significantly inhibited by GSK872 (Fig. 1d–f). IL-1β and IL-18 are important downstream molecular modulated by NLRP3 inflammasome pathway and acts as pivotal inflammatory factors in the LPS-induced ALI. IL-1β and IL-18 expression in lung tissue were apparently enhanced in ALI mice compared with sham mice. The selective RIP3 inhibitor GSK872 significantly inhibited IL-1β and IL-18 production as measured by ELISA (Fig. 1g, h). To further examine the effect of GSK872 on NLRP3 priming, NF-κB p65 and p-ERK were detected by western blot. However, the expression of NF-kB p65 and p-ERK was not changed with treatment of treatment of GSK872 (Additional file 1: Figure S1).

### Inhibition of RIP3 reduced lung injuries induced by LPS

**Fig. 2:**
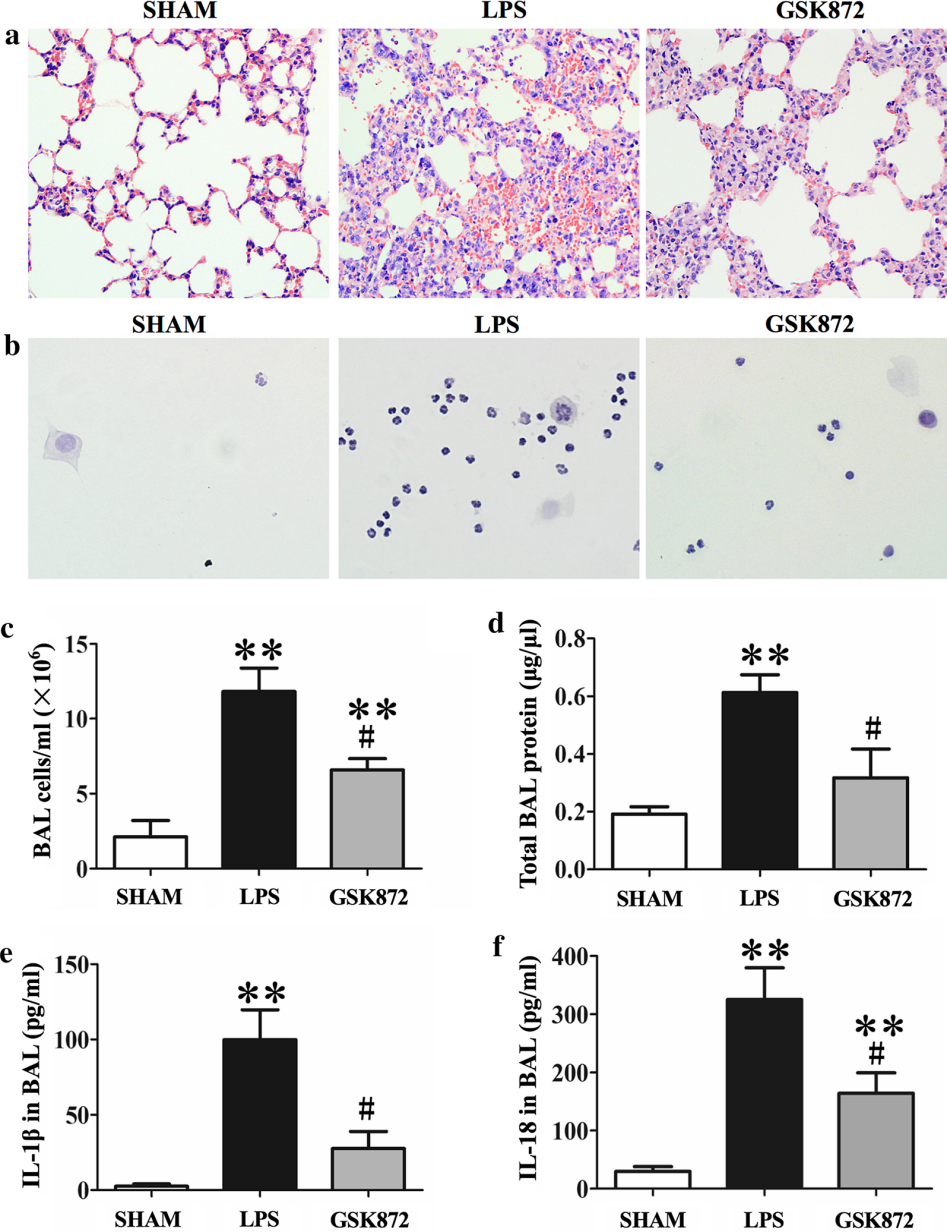
GSK872 decreased cell count IL-1β and IL-18 level in BAL fluid of LPS-induced ALI mice, as well as attenuated the histopathological injury. **a** HE staining of lung sections shows GSK872 treatment attenuated pulmonary injury and inflammatory cell infiltration in ALI mice. **b** HE staining of cytospun cells from BAL fluids. **c** Total cell counts of BAL fluids in Sham (PBS control), LPS and GSK872 (LPS + GSK872 treatment) groups. **d** Total protein level in BAL fluids. **e**, **f** IL-1β and IL-18 level in BAL fluid measured by ELISA. Each bar represents mean ± SEM (n = 6). **p < 0.01 versus sham mice; ^#^p < 0.05 versus LPS-induced lung injury mice

The effects of GSK872 on lung injury induced by LPS were investigated by histological examination with H&E staining. As shown in Fig. 2a, inflammatory cell infiltration was observed in the pulmonary interstitium of mice with LPS-induced ALI and was significantly reduced by GSK872 treatment. Cytospin cells from BAL fluids showed most of the infiltrated cells in the BALFs were neutrophils and macrophages. The numbers of these cells were decreased by GSK 872 treatment (Fig. 2b). To further investigate the effect of GSK872 on lung inflammation, BAL fluids were collected for cell counts, total protein, IL-1β and IL-18 level analysis. In normal control mice, there were very few cells observed in the BAL fluid and almost were alveolar macrophages. Cell number was significantly increased in the BAL fluids from mice with LPS-induced lung injury and neutrophil was the dominated cell type. In contrast, GSK872 treatment significantly reduced total cell number in the BAL fluid (Fig. 2c). Coincident with the cell counts, the total protein, IL-1β and IL-18 level in supernatants of BAL fluids was also elevated in the LPS-induced lung injury group, which was significantly reduced by GSK872 treatment (Fig. 2d–f).

### GSK872 inhibited NLRP3 inflammasome activation in lung interstitial macrophages (IMs) and CD 11b^+^ monocyte–macrophages/dendritic cells (M–M/DCs) cells in ALI

**Fig. 3:**
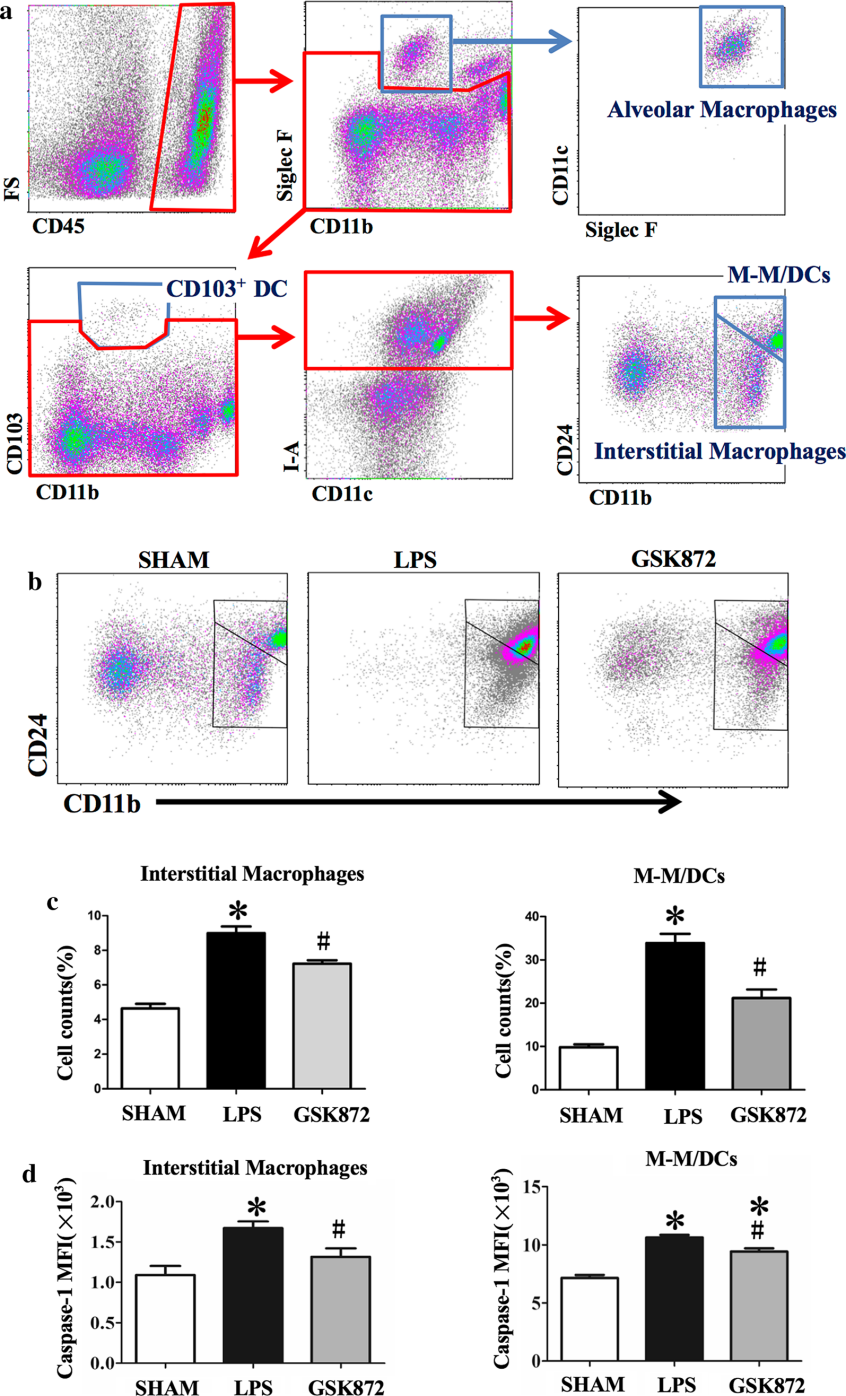
GSK872 reduced active caspase-1 level in lung interstitial macrophages (IMs) and CD45^+^I-A^+^CD11b^hi^CD24^+^ monocyte–macrophages/dendritic cells (M–M/DCs) of LPS-induced ALI mice. **a** Identification of CD45^+^SiglecF^+^CD11c^+^CD11b^lo^ alveolar macrophages, CD45^+^I-A^+^CD11b^+^CD24^lo^ IMs, CD45^+^I-A^+^CD11b^hi^CD24^+^ monocyte–macrophages/dendritic cells (M–M/DCs) and CD45^+^CD103^+^ CD11b^lo^ dendritic cells (CD103^+^ DCs) in the lung tissues of sham mouse. **b** IMs and M–M/DCs in Sham (PBS control), LPS and GSK872 (LPS + GSK872 treatment) groups. **c** Cell count percentage of IMs and M–M/DCs to the total cell counts determined by FACS. **d** Median fluorescence intensity (MFI) of active caspase-1 in IMs and M–M/DCs determined by FACS. Each bar represents mean ± SEM (n = 4). *p < 0.05 versus sham mice; ^#^p < 0.05 versus LPS-induced lung injury mice

Using the gating strategy as showed in Fig. 3a, CD45^+^I-A^+^CD11b^+^CD24^lo^ IMs and CD45^+^I-A^+^CD11b^hi^CD24^+^ cells (M–M/DCs) were the major subsets of cells in the lung of mice with LPS-induced ALI (Fig. 3a). The percentage of IM and M–M/DC was significantly increased in mice with ALI (Fig. 3b, c). Activated caspase-1 as detected by the AM FLICA Assay was significantly elevated in the IM and M–M/DC of LPS-induced lung injury group. This elevation was inhibited by GSK872 (Fig. 3d).

### GSK872 inhibited LPS-induced necroptosis in THP-1, a human monocytic cell line

**Fig. 4:**
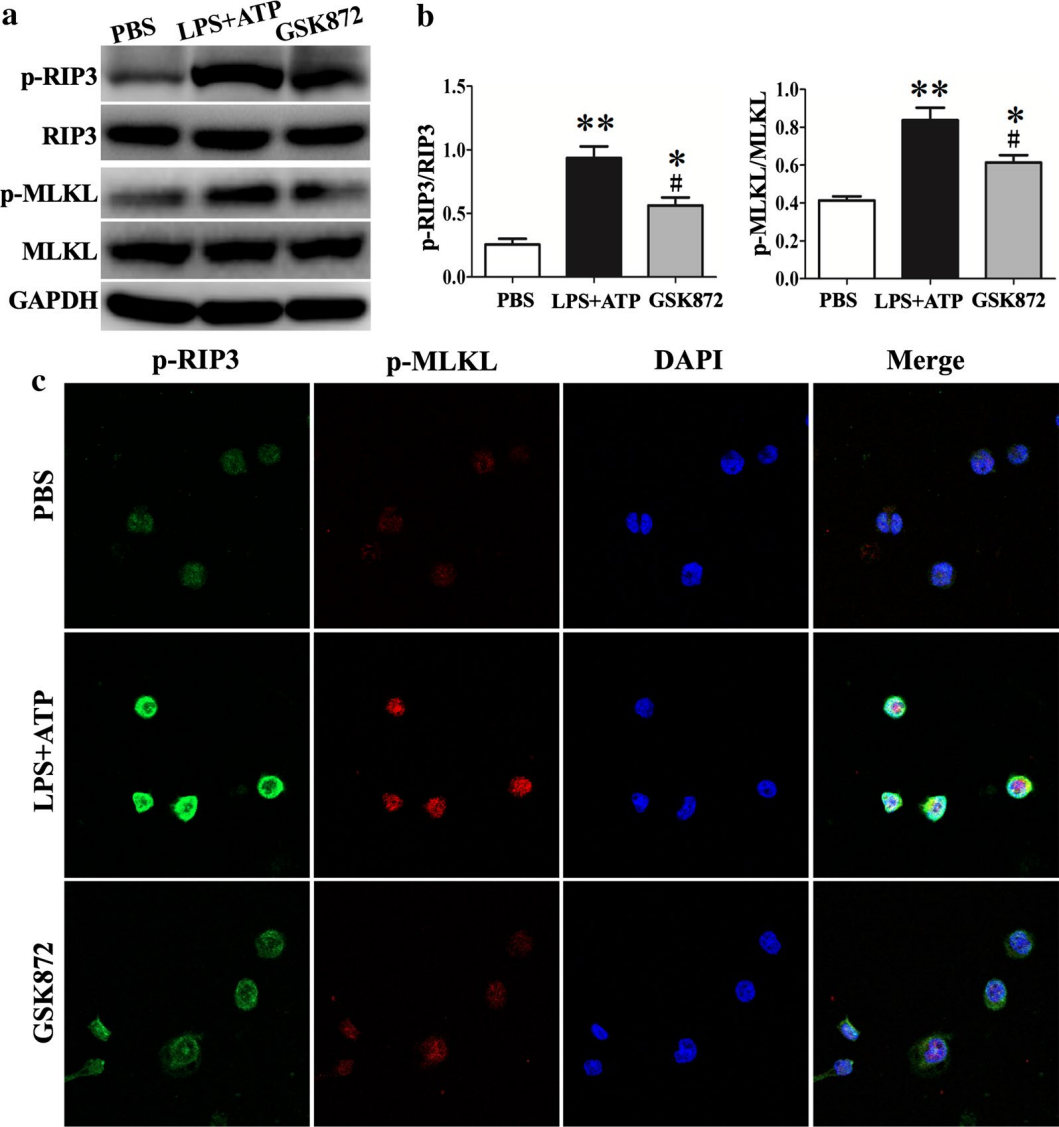
GSK872 inhibited LPS induced RIP3 mediated necroptosis in THP-1 cells. THP-1 cells were primed with 1 μg/ml LPS in the presence or absence of GSK872 (5 mM) for 4 h followed by stimulation with ATP (5 mM) for 1 h. **a** Representative western blot bands showed the protein expression of p-RIP3, RIP3, p-MLKL, MLKL and GAPDH. **b** Quantitative data showed protein expressions of p-RIP3 and p-MLKL normalized to the values of RIP3 and MLKL respectively. **c** Immunofluorescence showing the expression of p-RIP3 and p-MLKL. Each bar represents mean ± SEM (n = 4). *p < 0.05, **p < 0.01 versus PBS group; ^#^p < 0.05 versus LPS + ATP group

The above findings showed that IM and M–M/DC were the major sources of NLRP3 and caspase-1. To further investigate the RIP3–NLRP3 interaction in monocytes/macrophages, we used a human monocytic cell line THP-1 for the in vitro studies. Stimulation of THP-1 cells with LPS and ATP significantly elevated the expressions of p-RIP3 and p-MLKL. GSK872 inhibited RIP3 mediated necroptosis pathway as demonstrated by western bolt analysis (Fig. 4a, b). This inhibition was not complete but significant. Further immunofluorescence experiment showed that GSK872 significantly inhibited the expression of p-RIP3 and p-MLKL in LPS and ATP stimulated THP-1 cells (Fig. 4c).

### GSK872 inhibited NLRP3 inflammasome activation in THP-1 cells

**Fig. 5:**
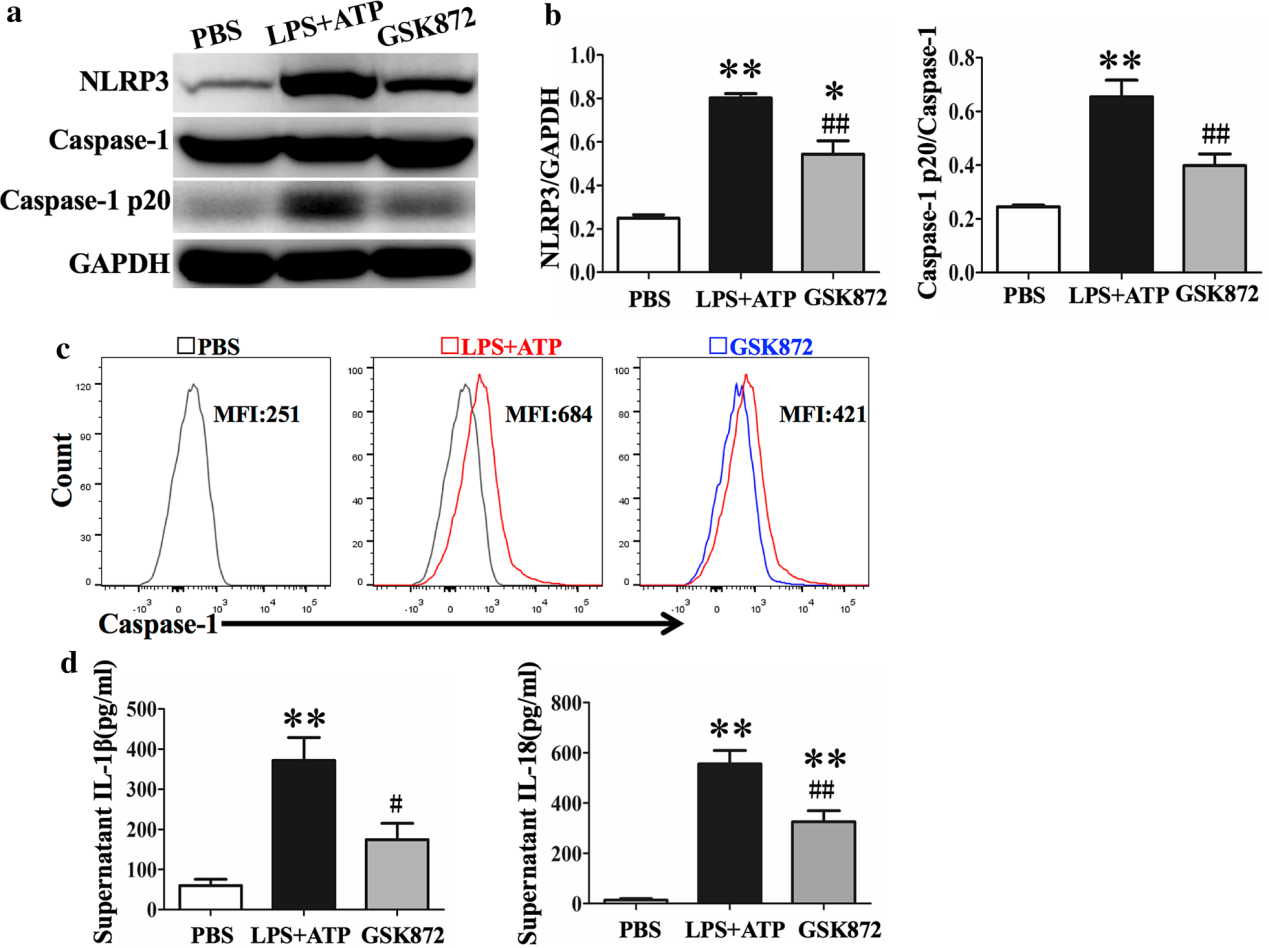
GSK872 inhibited LPS induced activation of NLRP3 pathway in THP-1 cells. **a** Representative western blot bands showed the protein expression of NLRP3, Caspase-1, Caspase-1p20 and GAPDH. **b** Quantitative data showed protein expressions of NLRP3 and caspase-1p20 normalized to the values of GAPDH and caspase-1 respectively. **c** Representative flow-cytometric histograms of active caspase-1 expression in THP-1 cell treated by PBS (grey line), LPS + ATP (red line) and LPS + ATP + GSK872 (blue line). **d** IL-1β and IL-18 level in supernatants from THP-1 cells determined by ELISA. Each bar represents mean ± SEM (n = 4). *p < 0.05, **p < 0.01 versus PBS group; ^#^p < 0.05, ^##^p < 0.01 versus LPS + ATP group

Stimulation of THP-1 cells with both LPS and ATP resulted in upregulation of NLRP3 and caspase-1p20 subunit. Pretreatment with GSK872 resulted in reduced this expression as demonstrated by western blot analysis (Fig. 5a, b). To further confirm the results of western blot, flow cytometry was used to analyze active caspase-1 in THP-1 cells. The results showed that GSK872 significantly reduced the level of active caspase-1 in THP-1 cells (Fig. 5c). The production of IL-1β and IL-18 in the supernatant was also significantly reduced by GSK872 treatment (Fig. 5d).

### Reduction of RIP1–RIP3 and RIP3–NLRP3 interactions in THP-1 cells by GSK872

**Fig. 6:**
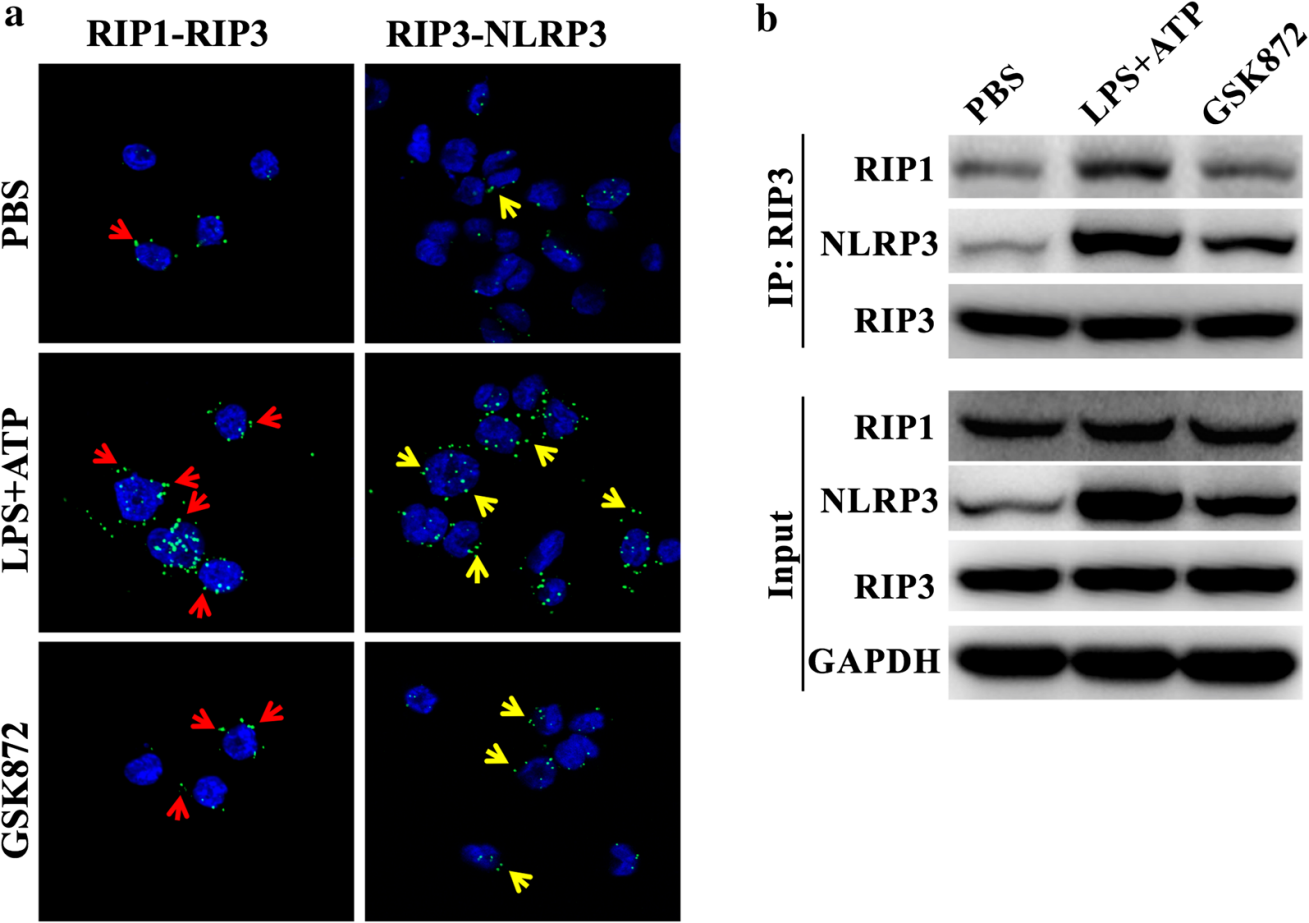
GSK872 inhibits LPS-induced NLRP3 activation by reducing RIP1–RIP3 and RIP3–NLRP3 interaction in THP-1 cells. **a** RIP1–RIP3 and RIP3–NLRP3 interactions in THP-1 cells were detected by Duolink^®^ In situ detection reagents and visualized by a fluorescence microscope. Signals were shown in green (red arrows indicated RIP1 and RIP3 interaction, yellow arrows indicated RIP3 and NLRP3 interaction) and the nuclei in blue. **b** RIP1–RIP3 and RIP3–NLRP3 interactions in THP-1 cells were further detected by immunoprecipitation study. RIP3 complexes isolated by immunoprecipitation from THP-1 cells were analyzed for RIP1, RIP3 and NLRP3 expression by western blot

Interactions between RIP3 and RIP1 and between RIP3 and NLRP3 were examined. RIP1–RIP3 and RIP3–NLRP3 interactions were detected using Duolink^®^ In situ detection Reagents, in which oligonucleotides were attached to the antibodies of both target proteins to form circular DNA, amplified and detected by fluorescent markers. As shown in Fig. 6a, RIP1–RIP3 and RIP3–NLRP3 interactions exhibiting green fluorescence were enhanced in THP-1 cells treated with LPS and ATP (middle panel of Fig. 6a), which were significantly reduced by GSK872 (lower panel of Fig. 6a). Protein interactions and their inhibition were confirmed by immunoprecipitation analysis. Antibodies to RIP3 brought down both RIP1 and NLRP3 (upper panel of Fig. 6b), documented the interaction between RIP3 with both RIP1 and NLRP3. The interactions between RIP3 and RIP1 and between RIP3 and NLRP3 were enhanced by treating THP-1 with LPS and ATP. GSK872 reduced the co-precipitation among RIP3 with RIP1 and NLRP3.

### Silencing RIP3 mRNA inhibited LPS-induced NLRP3 inflammasome activation

**Fig. 7:**
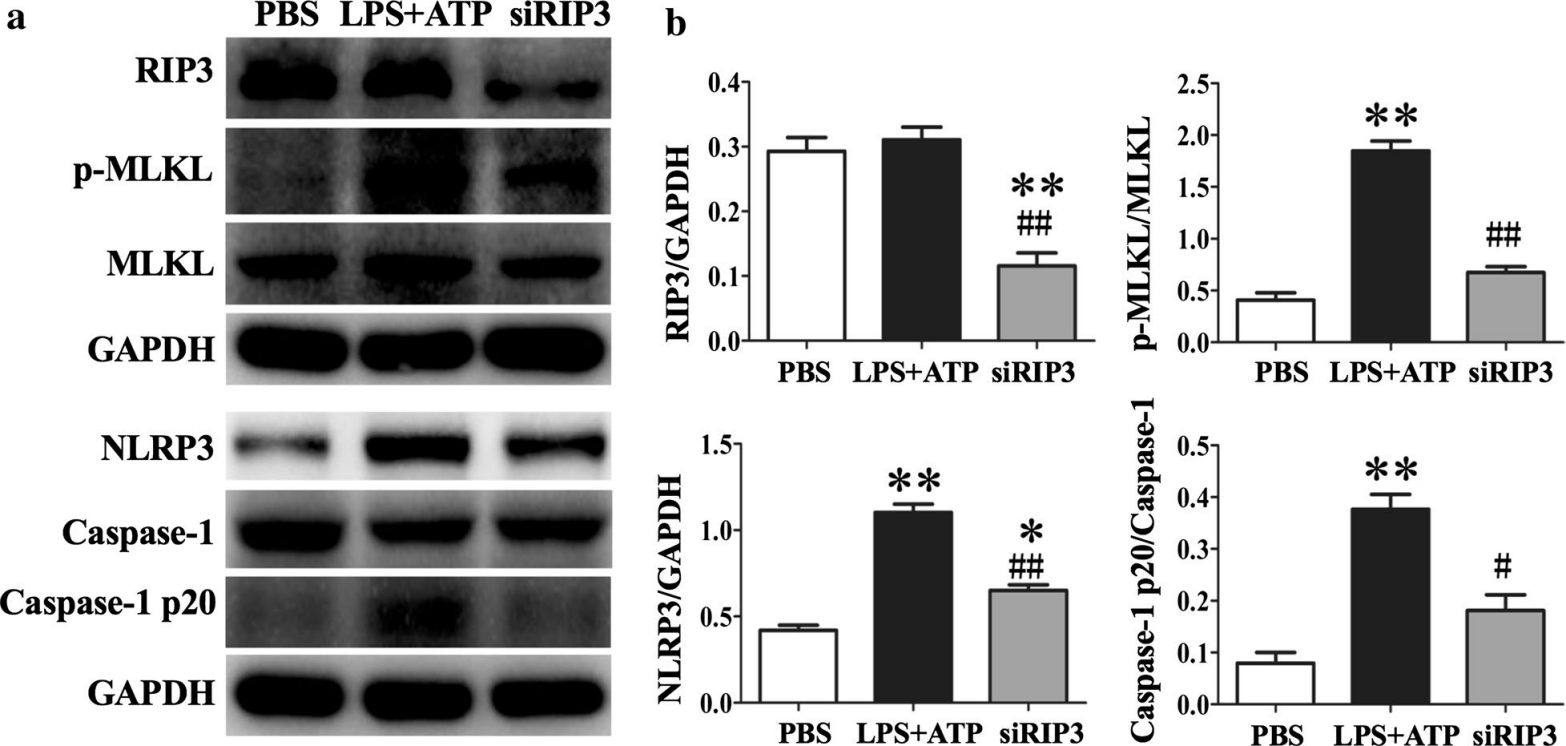
RIP3 silencing inhibited LPS-induced NLRP3 inflammasome activation in THP-1 cells. THP-1 cells were transfected with 100 nmol siRNA or scramble siRNA and cultured for 48–72 h before stimulation. **a** Representative western blot bands showed the protein expression of RIP3, p-MLKL, MLKL, NLRP3, Caspase-1, Caspase-1 p20 and GAPDH. **b** Quantitative data showed protein expressions of RIP3, p-MLKL, NLRP3, and caspase-1 p20 normalized to the values of RIP3, MLKL, GAPDH and caspase-1 respectively. Each bar represents mean ± SEM (n = 3). *p < 0.05, **p < 0.01 versus PBS group; ^#^p < 0.05, ^##^p < 0.01 versus LPS + ATP group

In order to further document RIP3 involvement in the activation of NLRP3, we knocked down RIP3 mRNA in THP-1 cells by the siRNA technology. As shown in Fig. 7, elevated expression of p-MLKL, NLRP3 and caspase-1p20 in THP-1 cells induced by LPS and ATP was significantly reduced when RIP3 was silenced.

## Discussion

RIP3, RIP1 and MLKL are essential components of necroptosis, a programmed cell necrosis that can cause inflammation and tissue damage. This process is the major cause of lung jury in staphylococcal pneumonia [14]. In LPS-induced systemic vascular inflammation, RIP3 has been implicated [15]. Directly relevant to our investigation, the necroptosis pathway was activated in LPS induced ALI [8]. This was shown in RIP3 knockout mice. LPS induced lung injury as well as the production of proinflammatory cytokines such as IL-1α/β, IL-6 and HMGB1 were decreased [8]. These findings suggest necroptosis elicit inflammatory responses during ALI. In contrast to these cited studies, our study showed that direct interaction of RIP3 with NLRP3 inflammasome play a major role in lung destruction in ALI. Thus, in addition to induction of necroptosis, RIP3 is a major player in the innate immune response in LPS induced ALI.

The mechanism by which RIP3 affects lung inflammation was not yet fully elucidated. Previous studies shown that NLRP3-caspase-1 inflammasome pathway participates in the inflammatory process in many diseases including ALI [2, 16, 17]. Recently, RIP3 was shown to interact with NLRP3 inflammasome resulting in IL-1β production and the induction of other inflammatory cytokines [18, 19]. In our study, GSK872 inhibited the phosphorylation of RIP3 and downstream MLKL as expected. In addition, GSK872 treatment extenuated the NLRP3-caspase-1 inflammasome activation as well as IL-1β production, indicating that RIP3 is engaged in the NLRP3 activation directly in LPS induced ALI.

Lung macrophages play major role in the pathogenesis of lung injuries irrespective of the mechanisms [10]. In this study, we found the percentage of interstitial macrophages was significantly increased in cells isolated from the injured lungs. The NLRP3 inflammasome was significantly activated in these isolated macrophages, suggesting that infiltrating macrophages were highly activated and responsible for production of proinflammatory cytokine such as IL-1β and IL-18. NLRP3 activation in infiltrating macrophages was significantly inhibited by GSK872, a specific inhibitor of RIP3. These results lead us to conclude that RIP3 was involved directly in the activation of NRLP3 in lung macrophages.

Our results have demonstrated that RIP3 mediated inflammatory process in LPS induced ALI through NLRP3 activation. In our in vitro study, the RIP3–NLRP3 interaction was significantly inhibited by RIP3 inhibitor GSK872, resulting in the inhibition of NLRP3 inflammasome activation which is independent of necroptosis. RIP3 can directly interact with NLRP3 to form RIP3–NLRP3 complex and lead to caspase-1 activation, resulting in IL-1β and IL-18 production. This mechanism has been confirmed by multiple approaches as detailed in our “Results” section.

The role of necroptosis in LPS-induced ALI requires further comments. Since RIP3 plays a crucial role in both necroptosis and NLRP3 inflammasome activation. Our studies provide new insight into the role of necroptosis in ALI. Undoubtedly necroptosis is associated with inflammatory responses in addition to NLRP3 inflammasome activation. The contribution of this important cellular process is beyond the scope of this paper.

## Conclusions

This study showed that RIP3 participates in the NLRP3 inflammasome activation in LPS-induced lung injury. This pathway is independent of RIP3 associated necroptosis. GSK872, a small molecule specific inhibitor of RIP3, inhibited significantly the inflammatory damages caused by LPS. Thus targeting the RIP3 signaling pathway would be a potential therapeutic strategy for treating ALI specifically and in other NLRP3 inflammasome mediated inflammatory conditions in general.

## Additional file

**Additional file 1: Figure S1.** GSK872 had no effect on NF-κB or ERK signaling in LPS/ATP stimulated THP-1 cells. Proteins were extracted from in LPS/ATP stimulated THP-1 cells and analyzed for phosphorylated-ERK (p-ERK) or total ERK (t-ERK) by western blotting. Nuclear proteins were obtained with a commercial nuclear extraction kit and subjected to western blotting for nuclear NF-κB p65. **A**, Representative western blot bands showed the protein expression of NF-κB p65, p-ERK and t-ERK. **B**, Quantitative data showed protein expressions of NF-κB p65 and p-ERK normalized to the values of fibrillarin and GAPDH, respectively. Each bar represents mean ± SEM (n = 3). **p < 0.01 versus PBS group.

## Abbreviations

ALI: acute lung injury
LPS: lipopolysaccharide
ARDS: acute respiratory distress syndrome
NLRs: nucleotide-binding oligomerization domain-like receptors
PRRs: pattern recognition receptors
ASC: apoptosis-associated speck like protein
RIPs: receptor interacting proteins
MLKL: mixed lineage kinase domain-like protein
