# Supplementary figures and images for: RIP3 dependent NLRP3 inflammasome activation is implicated in acute lung injury in mice
Source: J Transl Med. 2018 Aug 20;16:233. doi: 10.1186/s12967-018-1606-4 (PMC6102827; doi:10.1186/s12967-018-1606-4)

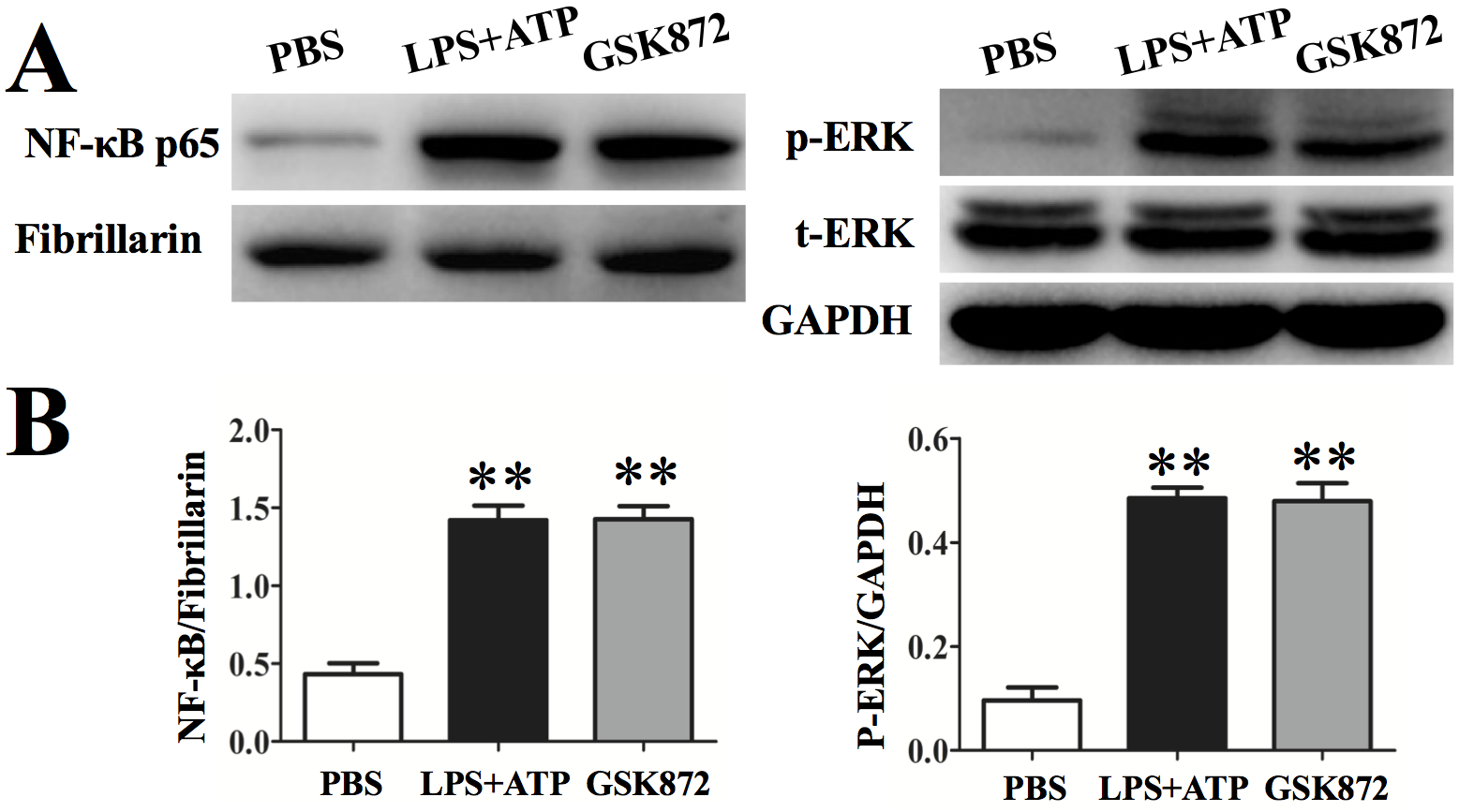

Supplement: Supplementary file 1 — Additional file 1: Figure S1. GSK872 had no effect on NF-κB or ERK signaling in LPS/ATP stimulated THP-1 cells. Proteins were extracted from in LPS/ATP stimulated THP-1 cells and analyzed for phosphorylated-ERK (p-ERK) or total ERK (t-ERK) by western blotting. Nuclear proteins were obtained with a commercial nuclear extraction kit and subjected to western blotting for nuclear NF-κB p65. A, Representative western blot bands showed the protein expression of NF-κB p65, p-ERK and t-ERK. B, Quantitative data showed protein expressions of NF-κB p65 and p-ERK normalized to the values of fibrillarin and GAPDH, respectively. Each bar represents mean ± SEM (n = 3). **p < 0.01 versus PBS group. [file 12967_2018_1606_MOESM1_ESM.tiff]
